# Initial Validation of the Coach-Athlete Relationship Questionnaire in a Sample of Portuguese Athletes

**DOI:** 10.1177/00315125241254437

**Published:** 2024-05-17

**Authors:** Ana Pinho, Diogo Monteiro, Miguel Jacinto, Rui Matos, Filipe Rodrigues, Nuno Amaro, Pedro Teques, Teresa Fonseca, Raúl Antunes

**Affiliations:** 1 Polytechnic University of Leiria, Leiria, Portugal; 2 Research Center in Sport, Health, and Human Development (CIDESD), Vila Real, Portugal; 3 N2i, Polytechnic of Maia, Maia, Portugal; 4 Polytechnic Institute of Guarda (IPG), Guarda, Portugal; 5 Centro de Investigação Formação Inovação e Intervenção em Desporto (CIFI2D), Porto, Portugal

**Keywords:** closeness, commitment, complementarity, enjoyment, invariance

## Abstract

Our primary objectives in this study were to translate and provide psychometric support for the Coach Athlete Relationship Questionnaire (CART-Q) Portuguese version, assess its invariance across sex, and explore its nomological validity in relation to enjoyment. Our sample participants were 470 athletes (226 females, 244 males) aged between 16 to 39 years from various individual and team sports. We found that the translated Portuguese version of the CART-Q exhibited satisfactory test-retest reliability and can serve as a reliable tool for evaluating the core constructs of the coach-athlete relationship – closeness, commitment, and complementarity. Moreover, this instrument showed evidence of nomological validity through significant positive correlations between its underlying factors and athletes' enjoyment with their sport. The proposed model for explaining item variance was also found to be invariant between male and female respondents. We recommend further use of this instrument in research and practical applications.

## Introduction

Recent research in the sports context has explored how the behaviors and attitudes of various athletes affect their performance and reactions during sports practice (Roberts, 2012). Coaches are key influencers in this arena, shaping athletes' physical and psychological development and creating optimal learning conditions (Duda, 2013; Short & Short, 2005). In addition to effective communication skills, coaches must have knowledge of training principles and methods of sports-related assessment (Jowett, 2003; Short & Short, 2005). They must also have competence in establishing quality working partnerships with their athletes, as this relationship has been seen as a significant influence for promoting the ongoing development of athletes' physical and psychosocial abilities (Jowett, 2005). The training process involves interpersonal situations in which the coach and athletes must engage with one another to maximize their potential, regardless of the type of sport, level, age, or sex of the athletes and coach (Jowett, 2005). When the coach-athlete relationship is effective, the athlete has the requisite conditions in which to acquire knowledge, experiences, and skills to optimize their performance (Philippe & Seiler, 2006). An effective relationship promotes feelings of security in difficult situations (e.g., injuries), guides the athlete through transitional situations (e.g., end of a career), and supports the athlete in moments of emotional crisis (e.g., failing in a major competition), all of which are essential factors for achieving optimal performance (Yang & Jowett, 2012). The quality of the interaction between athletes and coaches has been recognized as a determining factor in the athlete's sporting performance, due to its complexity and its impact on the behavioral, cognitive, and emotional aspects of sport for both player and coach (Monteiro, Pelletier, et al., 2018, Monteiro, Teixeira et al., 2018). This relationship is characterized by high levels of interdependence that can have either a positive or negative influence on performance, depending on how this interdependence is experienced (Jowett & Poczwardowski, 2007).

The coach-athlete relationship reflects the interconnection of emotions, thoughts, and behaviors between coaches and athletes (Jowett, 2007), and it significantly contributes to athletes' success and satisfaction (Jowett & Cockerill, 2002). Coaches play a fundamental role in athletes' development, as coaches use their knowledge and experience to aid both personal an athletic growth (Jowett, 2005; Jowett & Poczwardowski, 2007). This relationship is operationalized through closeness, commitment, and complementarity in a universal way that is represented by the 3C's model as described below (Jowett, 2007), with a long lasting influence on an athlete's life and career (Appleton & Duda, 2016; Jowett & Cockerill, 2002; Jowett & Poczwardowski, 2007).

### The 3C'S Model

The 3C's conceptual model of the coach-athlete relationship is comprised of (a) closeness (affective - respect, trust), (b) commitment (cognitive - long-term intentions), and (c) complementarity (behavioral - cooperative actions). Closeness reflects the emotional significance of the coach-athlete relationship, including contributions to emotional bonds through respect, admiration, and appreciation. Commitment is the cognitive aspect of this relationship, involving a long-term orientation and feelings of dependence. Complementarity, the behavioral aspect, pertains to cooperative behaviors, such as receptivity and friendliness (Jowett, 2005, 2007; Jowett & Nezlek, 2012).

The Coach Athlete Relationship Questionnaire (CART-Q) was developed to assess the athletes' perception of the quality of the coach-athlete relationship, as it aligns with the components of the 3 C's model – Closeness, Commitment, and Complementarity (Jowett & Ntoumanis, 2004). For closeness, the CART-Q measures feelings, for commitment, the CART-Q measures thoughts, and for complementarity, the CART-Q measures behaviors (Jowett, 2007, Jowett et al., 2017). Numerous qualitative studies have explored the coach-athlete relationship, through a principle focus on these 3 C's, and this literature has generally supported these constructs as universally applicable (Balduck & Jowett, 2010). The original validation study of the CART-Q, conducted by Jowett and Ntoumanis (2004), supported the measure's reliability and validity, through its association with interpersonal satisfaction, demonstrating the usefulness of this tool for studying coach-athlete relationships. In this original validation research, however, doubts were raised about the CART-Q’s discriminant validity; factor combinations approached or exceeded 1 in their 95% confidence intervals. The authors suggested a possible overlap between the three refined factors (Jowett & Ntoumanis, 2004). However, they emphasized that, despite high factor correlations, the three factors of the CART-Q should be considered separate dimensions.

In follow-up research with a Belgian version of the CART-Q all factor loadings were high and statistically significant, confirming its original factor structure (Balduck & Jowett, 2010). A validation study for a Chinese version of the CART-Q revealed strong relationships between Closeness and Commitment, both in their direct connectivity and from a meta-perspective. The direct perspective portrays the perception of personal emotions, thoughts, and actions of one an athlete in relation to the coach (e.g., "I have positive feelings toward my coach"). In contrast, the meta-perspective illustrates the athelte’s perception of how the coach feels, thinks, and acts toward the athlete (e.g., "My coach has positive feelings toward me"). Yang and Jowett (2012) asserted that the 3 C's can be considered separate but interrelated dimensions, and Yang and Jowett (2012) later aimed to standardize the CART-Q across seven countries and cultures. While they found the CART-Q to be valid, they observed cross-cultural differences on two items, suggesting that interpretations and evaluations might vary, based on cultural contexts. In a more recent study, investigators working with a French version of the CART-Q found that Commitment was a stronger predictor of respondents’ mental preparation and goal setting, while Closeness was more predictive of their technical skill training (Nicolas et al., 2017). Athletes showing greater commitment tended to be more loyal, leading coaches to work more closely with them to strengthen their psychological well-being and achieve their goals (Nicolas et al., 2017).

The sex of the athlete can influence their perceptions in the coach-athlete relationship (Jowett & Clark-Carter, 2006), and this variable has been a factor in CART-Q results. For example, LaVoi (2007) found that women reported higher levels of closeness than men, indicating a qualitative difference in how women and men perceive their relationships with the coach. Jowett and Clark-Carter (2006) found no significant sex differences in closeness, but they observed higher levels of commitment and complementarity among female athletes and their coaches than among male athletes and coaches. Female coaches tended to have more similar perceptions of commitment compared to coaches of male athletes. Female athletes also reported higher training satisfaction and performance (Jowett & Clark-Carter, 2006). Jowett and Nezlek (2012) supported these findings, indicating that British female athletes perceived more similarity in their responses to those of their coaches, suggesting a stronger coach-athlete bond. A recent study by Sanz-Fernández et al. (2023) revealed that women, particularly professional athletes, scored higher in emotional dimensions (e.g., commitment and closeness). The sex difference appeared to be moderated by the athlete’s level of sports involvement, possibly due to the prevalence of male amateur coaches. A validation study of direct and meta-perspective versions of the CART-Q translated to Arabic showed significant sex differences in satisfaction and CART-Q scores. Female athletes had significantly higher scores in all subdimensions, likely influenced by cultural norms and female coaches in Kuwait (Ahmad et al., 2021). Hence, when assessing cognitive constructs such as perceived interpersonal behaviors of athletes’ regard to coaches, Rodrigues et al. (2020) emphasized the importance of conducting research within specific cultural and contextual frameworks. They argued that scales validated in one context should not be applied in another context without proper validation. Cid et al. (2022) highlighted methodological limitations associated with the non-adaptation and validation of questionnaires, which could compromise the results of various studies in the sport context. The CART-Q appears suitable for use in sports settings in different cultures and languages but since it has not been validated for the Portuguese sport population, an analysis of its validity, reliability, and invariance for sex is also needed in this cultural context.

### The Association Between the Coach-Athlete Relationship and Both Enjoyment and Performance

Enjoyment in sports, defined as a positive emotional response encompassing pleasure and amusement (Scanlan & Simons, 1992), plays a central role in boosting athletes ‘engagement and commitment to sports, as is highlighted in the Sport Commitment Model (Scanlan et al., 1993). Young athletes often participate in sports primarily for enjoyment (Balish et al., 2014; Crane & Temple, 2015; Monteiro, Cid et al., 2017a; Monteiro, Nunes et al., 2017b), and their experience of joy and satisfaction encourages their long-term commitment (Balish et al., 2014; Scanlan et al., 1993). Enjoyment aligns with intrinsic motivation, and it encompasses joy, pleasure, satisfaction, challenge, and excitement (Ryan & Deci, 2017). Recent research has confirmed that the pursuit of enjoyable and pleasurable experiences in sports is a primary motivator for sports participation, while the absence of this element is a key factor in sports dropouts (Balish et al., 2014; Crane & Temple, 2015; Monteiro et al., 2018a). Enjoyment has consistently emerged as a strong predictor of the intention to continue sports participation (Teixeira et al., 2020).

### Present Study

Our main purpose in this study was to translate and validate the CART-Q (Jowett & Ntoumanis, 2004) in a sample of Portuguese athletes. We expected this instrument to facilitate various investigations of significant relevance to coaches in their interactions with Portuguese athletes. In full, we had three objectives in this research: (a) to test the psychometric proprieties of the CART-Q in a sample of Portuguese athletes; (b) to assess response invariance between male and female athletes; and (c) to explore the nomological validity of the CART-Q constructs by testing its association with enjoyment. We expected the newly adapted version of the instrument to provide coaches with reliable indicators of the quality of their relationships with athletes, a factor linked to the athletes’ increased satisfaction and enhanced sports performance (Jowett & Shanmugam, 2016; Monteiro et al., 2018a, 2018b). As prior literature has revealed sex differences, an understanding of these distinctions was expected to help others tailor interventions for specific research questions. Analyzing how the coach-athlete relationship factors might differ by sex was expected to contribute to improved sports performance (Jowett, 2008). Better understanding CART-Q associations with enjoyment should assist efforts to increase athletes’ sports participation and identify the main factors related to sports dropout (Crane & Temple, 2015; Jowett & Clark-Carter, 2006).

## Method

### Participants

We conducted an a priori power analysis using an online calculator (Soper, 2024), considering the following assumed parameters: an anticipated effect size of .2, statistical power of .8, three latent variables, 11 observed variables, and statistical significance of .05. The results of this calculation suggested an estimated minimum sample size of 296 participants. We estimated a need for an additional 10% to cover the risk of outliers and random missing values, and we concluded that we would need a total of at least 326 participants.

Final participants were 470 athletes (226 females, 144 females) from various individual and team sports (e.g., soccer, swimming, athletics, triathlon, gymnastics, basketball, volleyball, hockey) who were aged between 16 and 39 years (*M* = 22.05; *SD* = 7.29). Their number of training sessions per week ranged from 1 to 9 sessions (*M* = 4.28; *SD* = 1.97), and the duration of sport experience with the same coach varied from 1 to 26 years (*M* = 4.06; *SD* = 4.03). The instrument was answered at the end of one of the training sessions and lasted approximately 12 minutes.

### Ethical Considerations

We secured approval for this research protocol from the Ethics Committee (CE/IPLEIRIA/26/2021). All procedures adhered to the principles of the Helsinki Declaration (World Medical Association, 2013). All participants were informed about all the details and objectives of the study, including the assurance of their anonymity and confidentiality, which was clearly outlined in the questionnaire. We first contacted club presidents to outline the study's objectives and seek their permission for its implementation with their athletes. Subsequently, we reached out to the head coaches of each club, to whom we again explained the study's purpose and obtained their consent to administer the instruments to their athletes. Parents/legal guardians of all underage athletes provided informed consent for these minors’ participation in this study. All adult athletes in this study personally signed informed consent. The first authors of the study also explained the objective of the study to all participants who completed the study protocol before a training session in the absence of their coach.

For the test-retest analysis (see section below), three clubs were conveniently selected for their athletes to complete the same questionnaire at two different time points (50 athletes, *M* = 23.92; *SD* = 7.21). However, the data from this test-retest sample were not included in the total sample of 470 due to the questionnaire undergoing final revision stages. The informed consent form was completed by the adult athletes and by the parents/guardians of the minors. Therefore, although all participating athletes filled out the CART-Q under the same conditions, those athletes included in the test-retest sample were not recruited for the final sample.

### Procedures

For the translation and adaptation of the CART-Q instrument from its original English language version into Portuguese, we followed methodological procedures recommended by Vallerand (1989) and endorsed by Banville et al. (2000). Specifically, we followed these steps: (a) We conducted an initial translation with the assistance of three translators who were proficient in the English and Portuguese languages; (b) four experts independently evaluated the initial Portuguese version (First Assessment Panel); (c) four other experts (Second Assessment Panel) collectively examined all the items until they reached a consensus on item wording; (d) we administered this third version of the questionnaire to 50 independent athletes to assess item clarity and accuracy, with four weeks interval (Pilot Study); (e) two Portuguese language professors conducted a final review of the Portuguese version of the CART-Q to ensure correct syntax, spelling, and grammar were correct\ (Final Version).

### Measures

We used our translated Portuguese version of the CART-Q for measuring closeness, commitment, and complementarity within coach-athlete relationships with our athlete respondents. This adapted CART-Q was comprised of 11 items, categorized into the following factors: Closeness (3 items: e.g., “I like my coach.”), Commitment (4 items: e.g., “I feel close to my coach”), and Complementarity (4 items: e.g., “When I am coached by my coach, I feel at ease”). Participants rated these items on a Likert-type scale from 1 ("completely disagree") to 7 ("completely agree"), aligning conceptually with the 3C's model for coach-athlete relationships (Jowett, 2007). Previous studies (e.g., Yang & Jowett, 2012) have affirmed the validity and reliability of this method for evaluating the coach-athlete relationship constructs.

Perceptions of the degree of enjoyment was assessed using an instrument composed of eight items (e.g., “It is fun”) answered on a 7-point Likert scale ranging from 1 (“Totally disagree”) to 7 (“Totally agree”) based on the study of Monteiro et al. (2018a, 2018b). Other studies have confirmed the validity and reliability for assessing enjoyment in the sports context (Teixeira et al., 2020).

### Statistical Analysis

#### Test-Retest (n = 50)

We assessed the temporal stability of the CART-Q by following the recommendations of various authors (Banville et al., 2000; Cid et al., 2022). We used a two-way mixed effect, absolute agreement, single rater Intraclass Correlation Coefficients (ICC [2,1]) as proposed by several authors (Berchtold, 2016; Koo & Li, 2016). The ICC was interpreted as: poor <.50, moderate = .50 to .74, good = .75 to .90, and excellent >.90 (Koo & Li, 2016). Therefore, we employed the Intraclass Correlation Coefficient (ICC) to evaluate the reliability of responses to the Portuguese version of the CART-Q, with values above .70 deemed acceptable (Hair et al., 2019). The time interval between administrations of the instrument was four weeks, as suggested by several authors (Cid et al., 2022; Vallerand, 1989). Internal consistency of the factors was assessed using Cronbach's Alpha, and values above .70 were considered acceptable (Hair et al., 2019). This analysis was conducted using the Statistical Package for the Social Sciences (SPSS, Version 27, IBM Corp, Armonk, NY).

#### Factor Analysis (n = 470)

To assess the factor structure of this version of the CART-Q, we performed Exploratory Structural Equation Modeling (ESEM) following the recommendations of several authors (Marsh et al., 2014; Morin et al., 2013). We used the maximum likelihood robust estimator. Participants with missing data values that exceeded 5% of the instrument(s) were excluded from further analysis, in line with guidelines from Enders (2010). We used oblique target rotation procedures to specify an assumed model (Browne, 2001). In this study, we defined factors in a manner akin to the confirmatory factor models, allowing for cross-loadings to be estimated without constraints, although they were pushed to be close to zero. The ESEM analysis evaluated the three-factor correlations of the CART-Q Portuguese version for model fit, factor loadings, convergent and discriminant validity, and internal consistency. Model fit was assessed using traditional incremental and absolute fit indices, including CFI, TLI, SRMR, and RMSEA, along with their respective 90% confidence intervals (CI). The recommended cutoff values for these indices, as recommended by various authors (e.g., Hair et al., 2019), were adopted: CFI and TLI ≥ .90, and SRMR and RMSEA ≤ .80. Chi-square statistics are frequently employed to evaluate the fit of measurement models. Yet, as their sensitivity to sample size and model specifications can pose challenges (Hair et al., 2019), they are reported for transparency but not examined. Factor loadings above .50 were considered acceptable, indicating at least 25% of the latent factor variance (Hair et al., 2019).

We determined convergent validity using the average variance extracted, with acceptable values equal to or greater than .50, while discriminant validity was established when the squared correlations between factors were less than the extracted average variance for each factor (Fornell & Larcker, 1981). Lastly, internal consistency was evaluated through composite reliability (Raykov, 1997), with acceptable values of ≥ .70 (Hair et al., 2019). Analyses were conducted in MPLUS v.7.3 (Múthen & Múthen, 2010–2018).

#### Multigroup Analysis (n = 246 female; *n* = 224 male)

We conducted invariance analysis to determine whether the CART-Q Portuguese version could be applied equally to respondents of both sexes by following the procedures recommended by Morin et al. (2015). This analysis involved different types of invariance: (a) Configural invariance, which ensures that respondents of both sexes receive the same number of manifest variables (i.e., items) per latent variables (i.e., factors); (b) metric invariance, which verifies that the factor loadings for the respective factors (Closeness, Commitment, and Complementarity) have the same meaning in both groups; (c) scalar invariance, confirming that the results are related solely to the latent trait level of the respondents, regardless of their group membership; (d) residual invariance, which affirms that item residuals are equivalent for different groups. We evaluated the invariance assumptions based on the following criteria: (i) The measurement model must demonstrate a good fit to the data in each of the samples; and (ii) differences between different types of invariances should be less than .01 (Marsh et al., 2010) for CFI and TLI, and less than .015 for SRMR and RMSEA (Chen, 2007; Cheung & Rensvold, 2002).

#### Nomological Validity (n = 470)

We performed structural equation modeling to examine the associations between the constructs within the CART-Q and the Physical Activity Enjoyment Scale. We confirmed the suitability of the structural model using the previously mentioned indicators. We also scrutinized the standardized direct effects, including their corresponding 95% confidence intervals, considering them significant when the confidence interval did not encompass 0 (Hayes, 2018).

## Results

Table 1 displays the respondent’s test-retest reliability values, with the ICC of the items ranging from .84 (item 2) to .90 (item 7). This range signifies that the translated CART-Q questionnaire items displayed commendable test-retest reliability, surpassing the accepted threshold of .70. Consequently, these results emphasize the temporal reliability of the instrument. Additionally, it's apparent that the three factors fundamental to the CART-Q exhibited robust internal consistency, with values exceeding .70.

**Table 1.** Test-Retest Item and Factor Analysis.

Items	Athletes (*n* = 50)			
	*M* (*SD*)	ICC	*p*	Alpha
Item 1 Pre-Post	5.84 (1.27) - 5.82 (1.22)	.86	<.001	
Item 2 Pre-Post	5.94 (1.19) - 6.01 (1.23)	.84	<.001	
Item 3 Pre-Post	6.26 (1.19) - 6.27 (1.18)	.89	<.001	
Item 4 Pre-Post	6.37 (1.17) - 6.36 (1.17)	.85	<.001	
Item 5 Pre-Post	6.37 (1.17) - 6.37 (1.17)	.87	<.001	
Item 6 Pre-Post	5.81 (1.41) - 5.78 (1.15)	.88	<.001	
Item 7 Pre-Post	6.52 (1.02) - 6.48 (1.01)	.90	<.001	
Item 8 Pre-Post	6.59 (.985) - 6.56 (.995)	.87	<.001	
Item 9 Pre-Post	6.42 (1.08) - 6.38 (1.01)	.86	<.001	
Item 10 Pre-Post	6.33 (1.07) - 6.28 (1.12)	.88	<.001	
Item 11 Pre-Post	6.40 (1.05) - 6.44 (.988)	.89	<.001	
Closeness	6.41 (1.02) - 6.39 (1.06)	.87	<.001	.94 - .94
Commitment	5.87 (1.15) - 5.84 (1.24)	.89	<.001	.86 - .87
Complementarity	6.40 (.967) - 6.36 (1.01)	.90	<.001	.93 - .93

*Note. M* = mean; *SD* = standard deviation; ICC = intraclass correlation coefficient; *p* = level of significance.

The three-correlated factors ESEM model exhibited a strong fit across samples. This is evident from the CFI and TLI values exceeding .90, while the SRMR and RMSEA values remaining below .08. See details reported in Table 2.

**Table 2.** Psychometric Properties of the Three Correlated Factors Analyzed in the ESEM Model for Athletes.

Model	*χ2*	df	CFI	TLI	SRMR	RMSEA	IC90%
Total sample (*n* = 470)	153.63	25	.979	.955	.020	.068	.031–.121
Female sample (*n* = 246)	178.41	25	.968	.949	.034	.069	.030–.124
Male sample (*n* = 224)	184.71	25	.961	.944	.046	.072	.047–.127

*Note.* χ2 = Chi-squared; df = degrees of freedom; CFI = comparative fit index; TLI = Tucker-Lewis Index; SRMR = standardized root means square residual; RMSEA = root mean square error of approximation; IC90% = 90% confidence interval.

Analysis of the three-correlated factors ESEM model revealed that the factor loadings were consistently above .50 and statistically significant in their respective factors for both athletes and coaches. Each factor explained a minimum of 25% of the variance within the latent factor, and no cross-loadings were observed. Factor loadings on the non-targeted factor were below .50 and differences in factor loadings between targeted and non-targeted factor were less than .15 indicating absence of cross-loadings. The three factors demonstrated convergent validity. However, it is important to note that problems related to discriminant validity emerged among the three factors, as the squared correlations between factors exceeded the average variance extracted value for each of them as displayed in Table 3. Specifically, commitment and closeness were significantly associated (r2 = 0.77), commitment and complementarity were significantly associated (r2 = 0.96), and closeness and complementarity were significantly associated (r2 = 0.76).

**Table 3.** Factor Loadings of the Three Correlated Factor Models: Convergent, and Discriminant Validity Analysis.

Items	Commitment	Closeness	Complementarity	CR	AVE
Commitment					
Item 1	.98**	−.06^ns^	−.07^ns^		
Item 2	.80**	.03^ns^	.08^ns^	.82	.62
Item 6	.52**	.34*	.04^ns^		
Closeness					
Item 3	.28*	.63**	.18^ns^		
Item 5	.17^ns^	.72**	.16^ns^	.74	.50
Item 8	.08^ns^	.79**	.20^ns^		
Item 9	.35*	.68**	.21^ns^		
Complementarity					
Item 4	.22*	.15^ns^	.77**		
Item 7	.09^ns^	.24^ns^	.79**	.72	.53
Item 10	.13^ns^	.38*	.71**		
Item 11	.16^ns^	.21^ns^	.63**		

*Note***.**λ = standardized factor loadings; CR = composite reliability; AVE = Average Variance Extracted; ns = not significant, *p < .05; **p < .01.

The three-correlated factors models were examined for sex invariance, and the measurement model exhibited good fit in both male and female samples (see Table 2). The proposed model demonstrated invariance between male and female athletes, meeting all invariance assumptions. The multigroup analysis confirmed this invariance from configural invariance to all nested models, with negligible differences in fit indices (ΔCFI and ΔTLI < 0.01; ΔSRMR and ΔRMSEA < 0.015), as presented in Table 4.

**Table 4.** Multigroup Analysis of the Three-Correlated Factors Model Between Sexes.

Model	*χ2*	CFI	∆CFI	TLI	∆TLI	SRMR	∆SRMR	RMSEA	∆RMSEA
Configural	61.812*	.949	-	.938	-	.062	-	.051	-
Weak	57.234*	.947	.002	.935	.003	.066	.004	.049	.002
Strong	77.334*	.946	.003	.932	.006	.067	.005	.050	.001
Strict	97.664*	.939	.010	.927	.011	.082	.020	.055	.004

*Note.* ∆ = differences; *p < .001.

The structural equation model considering the CART-Q constructs as independent variables and enjoyment as the dependent variable indicated a good fit to the data (χ^2^ = 239.09; *p* < .001; df = 68; CFI = .979; TLI = .968; RMSEA = .073 [CI90% .063, .084]; SRMR = .023). The standardized direct effects revealed positive and significant associations between the factors underlying the CART-Q and enjoyment: Closeness (β = .10; 95% CI [.041, .220]), Commitment (β = .25; 95% CI [.110, .432]), and Complementarity (β = .25; 95% CI [.321, .824]).

## Discussion

Our objectives in this study were to translate and validate the CART-Q Portuguese version, assess its invariance across sex and across coaches and athletes, and explore its nomological validity in relation to enjoyment and perceived performance. Our analysis of the three underlying factors of the CART-Q revealed satisfactory internal consistency values, with alpha values for each factor ranging from .86 to .94. These results indicated a high level of test-retest item and factor reliability, exceeding the threshold of .70, consistent with the results showed by several other authors examining different versions of this instrument (Balduck & Jowett, 2010; Jowett & Ntoumanis, 2004; Nicolas et al., 2017; Yang & Jowett, 2012).

The ESEM analysis confirmed the presence of three-correlated factors: Commitment, Closeness, and Complementarity. All items displayed significant factor loadings that exceeded .50, within their respective factors, underscoring their strong associations with the targeted factor (Hair et al., 2019). The average variance extracted (AVE) values, ranged from .50 to .62, meeting criteria for acceptability (Fornell & Larcker, 1981) and indicating that these items effectively captured distinct and relevant dimensions within each factor.

In contrast to prior research findings in which sex invariance was not established (e.g., Ahmad et al., 2021; Jowett & Clark-Carter, 2006; Jowett & Nezlek, 2012), we actively sought to address this issue and can confidently assert that, in this Portugese population, the model held equal validity and consistency for both male and female athletes, and that factors shared identical structures and interpretations in these two samples. This aspect is especially noteworthy because existing literature has already unveiled certain distinctions between men and women concerning these variables. For instance, the study conducted by Jowett and Clark-Carter (2006) revealed that, while there may be similarity in the aspect of closeness between male and female athletes, differences emerge in their perceptions of commitment and complementarity when comparing female athletes with their coaches to male athletes. Therefore, this instrument provides a valuable foundation for comparing, and contrasting sex-specific data, enhancing the practical utility of the results obtained. To our knowledge, this is the first study to test invariance of the athletes' version of the CART-Q. Through a meticulous multi-group analysis, we were able to confirm that all models achieved acceptable levels of invariance. This signifies that the factorial structures of our collected data remained consistent across genders, with the fit indices of the models indicating minimal disparities. Consequently, we can confidently conclude that the model holds equal validity and consistency for both men and women, and the factors share identical structures and interpretations. This aspect is especially noteworthy because prior literature unveiled certain distinctions between men and women concerning these variables.

Our findings indicate that the standardized direct effects of each CART-Q factor on athletes' enjoyment exhibit positive and statistically significant relationships. This implies that the dimensions of closeness, commitment, and complementarity are closely linked to heightened athlete enjoyment. These results underscore the significance of enjoyment within the context of athlete-coach relationships. Considering the importance of enjoyment as a pivotal determinant of sports adherence (Crane & Temple, 2015), as well as the outcomes of a study conducted by Jowett and Shanmugam (2016), which may elucidate the positive impact of a coach-athlete relationship on both athlete satisfaction and sports performance.

### Practical Implications

The availability of a validated adaptation of CART-Q for use with Portugese athletes now stands as a pivotal and psychometrically supported tool for future investigations to assess and analyze the quality of the coach-athlete relationship. The CART-Q presents an opportunity to unravel the factors that underlie the coach-athlete relationship. Its application may drive the development of new interventions to enhance this relationship, with implications for athletes ‘motivation, performance, and overall well-being (Jowett & Ntoumanis, 2004). These interventions, in turn, can be instrumental in promoting positive and successful sports experiences, consequently mitigating the issue of sports dropout (Jowett & Shanmugam, 2016; Monteiro et al., 2018a).

### Limitations and Directions for Further Research

An avenue for future research could be the examination of invariance between athlete respondents from individual and team sports, since there are past findings suggesting that these groups may show varying satisfaction with the coach-athlete relationship (Rhind & Jowett, 2012; Short & Short, 2005). Another limitation of this study is the restriction of these findings to Portuguese athletes. Additionally, since enjoyment plays a pivotal role in both initiating and sustaining engagement in sports (McCarthy et al., 2008; Monteiro et al., 2018a), exploring the associations between the factors underpinning CART-Q and enjoyment may be important to efforts to bolster athlete commitment and engagement in their respective sports. For prospective studies, we recommend adopting a longitudinal research design in which data are gathered at the beginning and conclusion of the sports season to study how constant emotional changes of both coaches and athletes (Balaguer et al., 2018) may affect the relationship over time. Lastly, it is advisable to consider the adaptation and validation of both the direct and meta-perspective versions of CART-Q. Through the incorporation of the 3C's + 1 model, it becomes feasible to identify dysfunctional relationships (Jowett, 2005) and delve deeper into the shared goals, values, and common beliefs that underpin the coach-athlete dynamic.

## Conclusion

This translated and psychometrically supported CART-Q Portuguese version displayed adequate reliability and validity and can now be used dependably for evaluating Portuguese athlete perspectives of the commitment, complementarity, and closeness in the coach-athlete relationship. These findings align with previous studies of other adaptations of the CART-Q that have also supported this instrument. Moreover, these data support the predictive validity of the CART-Q factors to reveal meaningful, positive correlations between closeness, commitment, complementarity with athletes' enjoyment. We emphasized the value of developing further studies of CART-Q with pertinent other variables of interest. We also identified some limitations of this study and gave ideas about ways to improve upon it.
